# Digital PCR quantification of ultrahigh ERBB2 copy number identifies poor breast cancer survival after trastuzumab

**DOI:** 10.1038/s41523-024-00621-x

**Published:** 2024-02-19

**Authors:** Pei Meng, Hina Dalal, Yilun Chen, Christian Brueffer, Sergii Gladchuk, Miguel Alcaide, Anna Ehinger, Lao H. Saal

**Affiliations:** 1 Division of Oncology, Department of Clinical Sciences Lund, Lund University Cancer Center, Skåne University Hospital Comprehensive Cancer Center, SE-22381 Lund, Sweden; 2 SAGA Diagnostics AB, Scheelevägen 2, MV406, SE-22381 Lund, Sweden; 3 Department of Genetics and Pathology, Laboratory Medicine, Region Skåne, Lund, Sweden

**Keywords:** Breast cancer, Predictive markers, Translational research, Tumour biomarkers, Breast cancer

## Abstract

HER2/ERBB2 evaluation is necessary for treatment decision-making in breast cancer (BC), however current methods have limitations and considerable variability exists. DNA copy number (CN) evaluation by droplet digital PCR (ddPCR) has complementary advantages for HER2/ERBB2 diagnostics. In this study, we developed a single-reaction multiplex ddPCR assay for determination of ERBB2 CN in reference to two control regions, CEP17 and a copy-number-stable region of chr. 2p13.1, validated CN estimations to clinical in situ hybridization (ISH) HER2 status, and investigated the association of ERBB2 CN with clinical outcomes. 909 primary BC tissues were evaluated and the area under the curve for concordance to HER2 status was 0.93 and 0.96 for ERBB2 CN using either CEP17 or 2p13.1 as reference, respectively. The accuracy of ddPCR ERBB2 CN was 93.7% and 94.1% in the training and validation groups, respectively. Positive and negative predictive value for the classic HER2 amplification and non-amplification groups was 97.2% and 94.8%, respectively. An identified biological “ultrahigh” ERBB2 ddPCR CN group had significantly worse survival within patients treated with adjuvant trastuzumab for both recurrence-free survival (hazard ratio, HR: 3.3; 95% CI 1.1–9.6; *p* = 0.031, multivariable Cox regression) and overall survival (HR: 3.6; 95% CI 1.1–12.6; *p* = 0.041). For validation using RNA-seq data as a surrogate, in a population-based SCAN-B cohort (NCT02306096) of 682 consecutive patients receiving adjuvant trastuzumab, the ultrahigh-ERBB2 mRNA group had significantly worse survival. Multiplex ddPCR is useful for ERBB2 CN estimation and ultrahigh ERBB2 may be a predictive factor for decreased long-term survival after trastuzumab treatment.

## Introduction

Overexpression of the ERBB2 oncogene, located on chromosome 17q12 and commonly referred to as HER2 (human epidermal growth factor receptor 2), was recognized decades ago as a key factor associated with poor outcome in primary breast cancer (BC)^1,2^. It is a member of the epidermal growth factor receptor (EGFR) family of receptor tyrosine kinases, functions to promote cell growth and proliferation, and is genomically amplified in ~15–20% of breast cancers^3^. The importance of HER2 amplification status as a prognostic marker in BC led to the development and approval of various antibody-based drugs targeting HER2 such as trastuzumab, which today remains the mainstay for treatment of ERBB2/HER2-amplified primary BC, and has cemented HER2 status as a vital treatment-predictive biomarker^4,5^. Although the clinical outcomes for patients with HER2-positive BC have dramatically improved due to trastuzumab and other anti-HER2 therapies, notwithstanding appropriate treatment, it remains that 15–20% of HER2-positive patients still experience relapse of the disease within 5 years^5–7^. Therefore, novel diagnostics tools to further stratify response in HER2-positive disease are highly desirable.

Clinical HER2 status workup, as recommended by national guidelines such as the 2018 American Society of Clinical Oncology and the College of American Pathologists (ASCO/CAP) HER2 guidelines, consists of a diagnostic algorithm employing immunohistochemistry (IHC) and in situ hybridization (ISH)^8^. Although part of the current gold standard, it is widely understood that these methods have certain limitations: IHC measures protein level, is semiquantitative and may have considerable inter-laboratory and inter-evaluator variability, and all IHC 2+ equivocal cases require an additional fluorescent-/silver-/chromogenic- ISH test for determining HER2 status definitively^9^. Additionally, some local and national guidelines recommend ISH verification of IHC 3+^10^. Compared to IHC, ISH-based tests are more labor intensive, time-consuming, and expensive, although the evaluations are more reproducible^11,12^. Both methods are susceptible to pre-analytical factors such as time to fixation and duration of fixation, and neither IHC nor ISH are truly quantitative. Importantly, dual-probe ISH methods ascertain CN of the chromosome 17 centromere and therefore can distinguish monosomy vs polysomy/co-amplification, revealing five possible test result scenarios: 95% fall into either group 1 classic HER2 amplified or group 5 classic HER2 non-amplified cancer, and the remaining 5% fall into either group 2 monosomy 17, group 3 co-amplification/polysomy 17, or group 4 borderline/equivocal^13^.

Digital polymerase chain reaction (dPCR) is a highly sensitive, precise, and accurate technology for absolute quantification of DNA copy number (CN). In contrast to quantitative PCR (qPCR), dPCR does not require concentration standards and the method has been shown to be highly linearly quantitative across many orders of magnitude. Although dPCR has been successfully employed for ERBB2 CN estimation and shown to be accurate for determination of HER2 status^14–20^, these studies had a number of limitations such as small sample size, no patient clinical outcomes, or used dPCR assays with only one reference region control probe.

The goal of this study is to develop an improved assay for rapid, reproducible, accurate, and cost-effective ERBB2 CN estimation, and to evaluate the relationship of a more accurate estimation of ERBB2 CN to the response to trastuzumab across a large breast cancer patient cohort.

## Results

### ERBB2 assay development

**Fig. 1:**
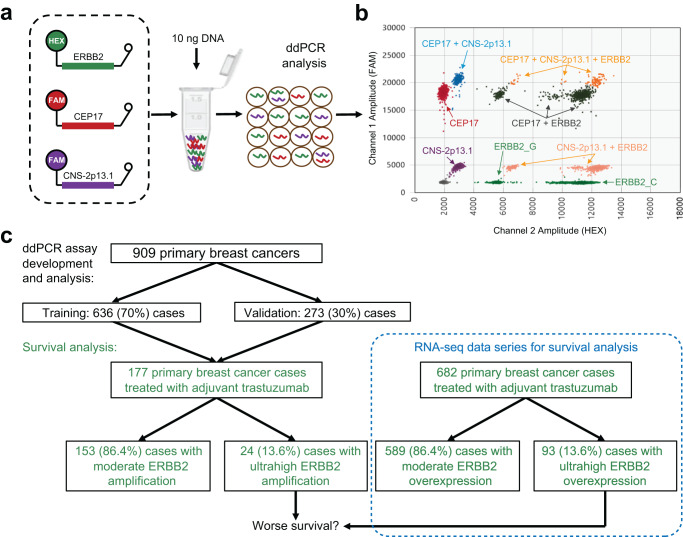
ERBB2 ddPCR multiplex assay and study workflow. **a** A multiplex ddPCR assay was developed to measure DNA copy numbers at three genomic regions, namely ERBB2 (at SNP rs1058808), CEP17, and a copy number stable region CNS-2p13.1. **b** Data analysis revealed distinct ddPCR signal clusters representing each region and ERBB2 allele. Performance on normal human genomes and SK-BR-3 ERBB2-amplified positive control is shown in Supplementary Figure 1. **c** A total of 909 primary breast cancer cases were evaluated by ddPCR, randomly divided into 70% (636 cases) as training group and 30% (273 cases) as validation group. Among the 909 cohort, 177 patients received adjuvant trastuzumab. Survival analysis was performed to investigate the prognostic value of ultrahigh ERBB2 CN in patients receiving trastuzumab, and the association was verified in an RNA-seq data cohort of 682 primary breast cancer patients who received adjuvant trastuzumab.

We designed and validated a novel single-reaction multiplex droplet digital PCR (ddPCR) assay to quantify two alleles of the single nucleotide polymorphism (SNP) rs1058808 within ERBB2 simultaneously with two reference control genomic regions, the chromosome 17 centromere (CEP17) and a breast cancer CN stable region located near cytoband 2p13.1 (CNS-2p13.1). To evaluate the assay, we utilized negative and positive control samples as well as 909 primary breast cancers highly selected to represent a diversity of HER2 statuses (Fig. 1).

The demographic and histopathologic data for the 909 BC cohort are presented in Table 1. The median age at diagnosis was 65 years (range, 25–95 years) and median tumor size was 19 mm (range, 1–220 mm). Nottingham histological grade Ι, ΙΙ and ΙΙΙ were 11.2%, 44.2% and 44.3%, respectively. From clinical records, 437 patients (48.1%) were HER2 IHC 0-1+, 236 patients (26.0%) were IHC 2+, and 236 (26.0%) were IHC 3+. Of these, 30 IHC 2+ cases and 230 IHC 3+ cases were clinically ISH positive, as were 4 IHC 0-1+ cases. Prior to evaluation, the 909 BC cohort was randomly divided into a training set of 636 cases, and a validation set of 273 cases.

**Table 1:** Study population clinical characteristics

	Sample Number (*n* = 909)
Patient age (years)	
Median (range, SD)	65 (25–95, 13.2)
Tumor size (mm)	
Median (range, SD)	19 (1–220, 13.8)
HER2 IHC status	
0-1+	437 (48.1%)
2+	236 (26.0%)
3+	236 (26.0%)
HER2 ISH status	
Amplified	264 (29.0%)
Normal	481 (52.9%)
n/a	164 (18.0%)
HER2 clinical status	
Positive	264 (29.0%)
Negative	644 (70.8%)
n/a	1 (0.1%)
ER status	
Positive	740 (81.4%)
Negative	168 (18.5%)
n/a	1 (0.1%)
PgR status	
Positive	629 (69.2%)
Negative	279 (30.7%)
n/a	1 (0.1%)
Ki67 status	
High	541 (59.5%)
Low	366 (40.3%)
n/a	2 (0.2%)
Nottingham histological grade	
G1	102 (11.2%)
G2	402 (44.2%)
G3	403 (44.3%)
n/a	2 (0.2%)
Lymph node status	
Negative	542 (59.6%)
1 to 3	239 (26.3%)
≥4	108 (11.9%)
n/a	20 (2.2%)

*ER* estrogen receptor alpha, *PgR* progesterone receptor.

The developed ERBB2 multiplex assay had distinct ddPCR signals with discrete clusters and excellent performance (Fig. 1b). Herein, “ERBB2 CN” and “CEP17 CN” are calculated using CNS-2p13.1 as reference, whereas “ERBB2/CEP17 ratio” is the quotient of ERBB2 CN by CEP17 CN (see Methods). Showing high reproducibility across replicate analyses on independent dates/operators of the Coriell NS12911 normal human control genomic DNA sample, close to the expected value of 2 the mean CNS-2p13.1 copies per genome was measured to be 1.98 with standard deviation (SD) of 0.04, the mean CEP17 copies was 1.89 (SD 0.06), and mean ERBB2 copies was 2.08 (SD 0.03) (Supplementary Fig. 1). Demonstrating similarly high reproducibility across repeat analyses on independent dates/operators of the well-characterized ERBB2-amplified SK-BR-3 breast cancer cell line, the mean CEP17 CN by ddPCR was 3.03 (SD 0.07) showing gain of one copy, the mean ERBB2 CN was 19.83 (SD 0.42), and the mean ERBB2/CEP17 ratio was 6.55 (SD 0.13). These results for SK-BR-3 positive control correspond well to orthogonal estimates for ERBB2 CN in SK-BR-3 from the literature, which range from 15 to 20 using methods such as FISH (CN 15) and massively-parallel sequencing (CN 15–20)^21–23^.

**Fig. 2:**
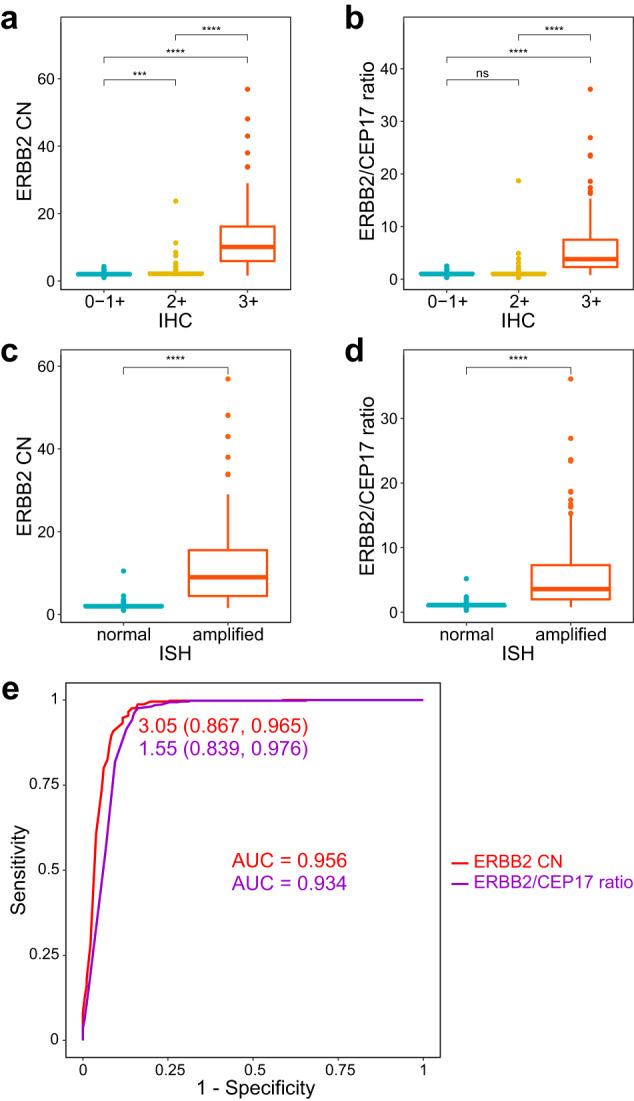
ERBB2 ddPCR measurements and clinical HER2 evaluations. Within the 636 training group, ERBB2 ddPCR multiplex assay measurements are significantly associated to (**a**, **b**) clinical HER2 IHC and (**c**, **d**) HER2 ISH results, and (**e**) receiver operating characteristic (ROC) analysis identifies optimal thresholds for defining amplification based on the ERBB2 ddPCR multiplex assay, using ERBB2 CN or ERBB2/CEP17 ratio. The boxplots present the median (center line), 25% and 75% quantiles (lower and upper bounds of the box), and outlier values (whiskers and datapoints). The optimal thresholds for each biomarker are given below the ROC curves, with corresponding sensitivity and specificity within parentheses. *P*-values calculated by *t*-test. ***p < 0.001; ****p < 0.0001; ns not significant, AUC area under curve, CN copy number, IHC immunohistochemistry, ISH in situ hybridization.

Likewise, the ERBB2 ddPCR multiplex assay had excellent performance across the 636 BC DNA samples of the training group. Within the training group, the mean CEP17 copies/genome was 1.85 (SD 0.73) and the mean CNS-2p13.1 copies/genome was 1.74 (SD 0.37), which improved slightly to 1.73 (SD 0.50) and 1.74 (SD 0.38), respectively, when considering only the cases clinically HER2 ISH non-amplified. The discrepancy from an expected value of 2 copies/genome is likely due to inaccuracies in the DNA concentration measurements by Nanodrop, which is used to load each ddPCR reaction. Nevertheless, ddPCR loading concentration is immaterial when calculating ERBB2 CN and ERBB2/CEP17 ratio. The ERBB2 ddPCR multiplex assay measurements correlated well with the clinical HER2 IHC and ISH diagnostic results. Both ERBB2 CN and ERBB2/CEP17 ratio was significantly associated to clinical HER2 IHC scores (all t-test *p*-values < 0.0001 for IHC 2+ vs 0-1+, IHC 3+ vs IHC 0-1+, and IHC 3+ vs IHC 2+; Fig. 2a, b) and HER2 ISH results (all *t*-test *p*-values < 0.0001 for ISH-amplified vs ISH-normal; Fig. 2c, d). Within the HER2 IHC 0-1+ (*n* = 310), IHC 2+ (*n* = 163), and IHC 3+ (*n* = 163) clinical groups, the mean ERBB2 CN was 2.00 (SD 0.39), 2.58 (SD 1.99), and 12.0 (SD 9.11), respectively. Correspondingly, the mean ERBB2/CEP17 ratio was 1.06 (SD 0.21), 1.27 (SD 1.46) and 5.71 (SD 5.32) for HER2 IHC 0-1+, 2+ and 3+ cases, respectively. Among the training set subgroup of clinically HER2 ISH-amplified cases (*n* = 180), the mean ddPCR ERBB2 CN was 11.33 (SD 9.04) and mean ERBB2/CEP17 ratio was 5.43 (SD 5.28). Lastly, and in line with non-amplified CN from the clinical records, among the subgroup of HER2 ISH-normal training cases (*n* = 345) the mean ERBB2 CN was 2.12 (SD 0.63) and mean ERBB2/CEP17 ratio was 1.10 (SD 0.30).

### HER2 status determination by ddPCR

Receiver operating characteristic (ROC) curve analysis was performed on the training group to determine the most appropriate cut-off values to define HER2 status based on either ddPCR ERBB2 CN or ERBB2/CEP17 ratio. This analysis revealed an area under the curve (AUC) of 0.956 for ERBB2 CN and 0.934 based on ERBB2/CEP17 ratio, with optimal thresholds at 3.05 and 1.55, respectively (Fig. 2e). The respective sensitivity and specificity were 86.7% and 96.5% for ERBB2 CN, and 83.9% and 97.4% based on ERBB2/CEP17 ratio. If using only ERBB2 CN or ERBB2/CEP17 ratio to determine HER2 status, non-classic HER2-positive cases may be misclassified including monosomy 17 and co-amplification of CEP17. Therefore, using a metric combining ERBB2 CN and ERBB2/CEP17 ratio, the sensitivity and specificity was 88.3% and 95.0% (Table 2). Finally, these thresholds determined on the training group were applied to the validation group and highly similar sensitivity, specificity, and accuracy was achieved (Table 2).

**Table 2:** Multiplex ERBB2 ddPCR assay performance metrics in training and validation

Cohort	Criterion	Threshold ^a^	Sensitivity	Specificity	Accuracy	PPV	NPV
Training group (*n* = 636)	ERBB2/CEP17 ratio	1.55	83.9%	97.4%	93.5%	92.6%	93.9%
	ERBB2 CN	3.05	86.7%	96.5%	93.7%	90.7%	94.8%
	Combination		88.3%	95.0%	93.1%	87.4%	95.4%
Validation group (*n* = 273)	ERBB2/CEP17 ratio	1.55	76.2%	97.9%	90.8%	94.1%	89.8%
	ERBB2 CN	3.05	83.3%	99.5%	94.1%	98.6%	92.6%
	Combination		84.5%	97.9%	93.4%	94.7%	92.9%

^a^Threshold is selected with maximum Youden’s index. *PPV* positive predictive value, *NPV* negative predictive value.

**Fig. 3:**
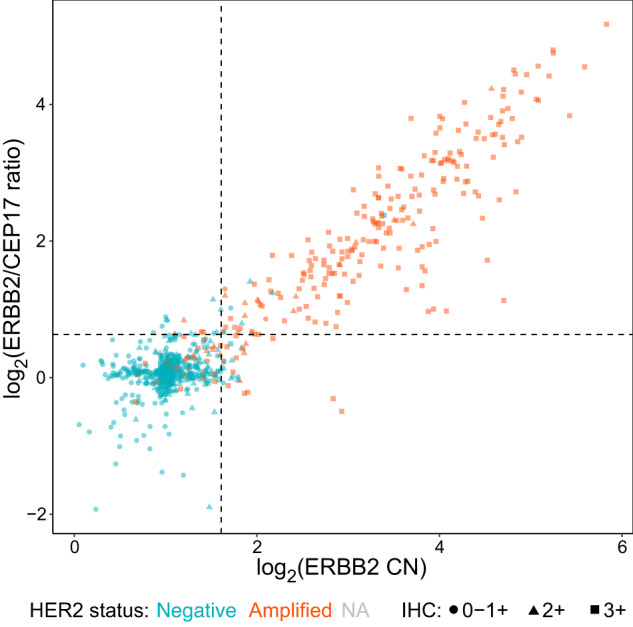
ERBB2 CN versus ERBB2/CEP17 ratio plot for all 909 breast tumors (training and validation groups combined), with the marker shape indicating clinical IHC score and the marker color indicating HER2 status from the clinical routine. The dotted lines indicate the optimal thresholds as determined by ROC analysis on the training group (see Fig. 2e). CN copy number, IHC immunohistochemistry, NA not available.

Among the complete series of 909 cases, we next investigated categorization using the thresholded ddPCR measurements into four groups similar to the 2018 ASCO/CAP HER2 guidelines: 217 (23.9%) were group 1 classic HER2 amplified, 14 (1.5%) were group 2 monosomy, 26 (2.9%) were group 3 co-amplification (polysomy), and 652 (71.7%) were group 5 classic HER2 non-amplified (Table 3); for this purpose, we did not assign any cases to an equivocal HER2 status group. Notably, 425/437 (97.3%) of IHC 0-1+ were ddPCR group 5 and only 4/437 (0.9%) were ddPCR group 1, only 26/236 (11.0%) of IHC 2+ were ddPCR group 1 or 3 (considered amplified), and 212/236 (89.8%) IHC 3+ were ddPCR group 1, 2, or 3 (considered amplified). To note, 61/909 (6.7%) of cases indicated gain of at least one CEP17 copy (CEP17 copies/genome >3). When compared to clinical HER2 ISH scoring of amplified vs normal, the positive predictive value for ddPCR group 1 classic amplification was 97.2% and the negative predictive value for ddPCR group 5 classic non-amplification was 94.8% (Table 3). Among the ddPCR-classified group 3 co-amplified cases, 15/26 (57.7%) had been evaluated ISH-amplified: 15/15 were IHC 2+ or 3+, whereas an additional 8/26 were also IHC 2+ or 3+ and thus may have been mis-classified clinically as HER2-negative. Among the ddPCR-classified group 2 monosomy cases, 9/13 (69.2%) were ISH-normal: 9/9 were IHC 0-1+ or 2+, whereas an additional 2/9 were also IHC 3+ and thus may have been mis-classified clinically as HER2-negative (Table 3). Finally, among the 909 cases, 6 cases were defined as ddPCR group 1 amplified (high ERBB2 CN and ERBB2/CEP17 ratio) but were clinically HER2-negative and did not receive anti-HER2 therapy (Fig. 3). Anecdotally, 3 of these 6 patients had recurrence of disease during follow up. These discrepant cases may represent clinical HER2 false-negatives due to technical factors during specimen preparation and routine pathological evaluation or biological factors such as intratumoral heterogeneity, with the subclone analyzed at diagnosis being HER2-negative but the specimen analyzed by ddPCR in this study containing a subclone with ERBB2 CN gain.

**Table 3:** Distribution of HER2 ddPCR status and HER2 status based on IHC and ISH

	Group 1: Classic HER2 amplified	Group 2: Monosomy 17	Group 3: Co-amplification (previously polysomy 17)	Group 5: Classic HER2 non-amplified	
	ERBB2 CN ≥ 3.05 & ERBB2/CEP17 ≥ 1.55 (*n* = 217)	ERBB2 CN < 3.05 & ERBB2/CEP17 ≥ 1.55 (*n* = 14)	ERBB2 CN ≥ 3.05 & ERBB2/CEP17 < 1.55 (*n* = 26)	ERBB2 CN < 3.05 & ERBB2/CEP17 < 1.55 (*n* = 652)	Totals (*n* = 909)
IHC					
0-1+	4 (1.8%)	5 (35.7%)	3 (11.5%)	425 (65.2%)	437 (48.1%)
2+	16 (7.4%)	7 (50.0%)	10 (38.5%)	203 (31.1%)	236 (26.0%)
3+	197 (90.8%)	2 (14.3%)	13 (50.0%)	24 (3.7%)	236 (26.0%)
ISH					
Amplified	211 (97.2%)	4 (28.6%)	15 (57.7%)	34 (5.2%)	264 (29.0%)
Normal	5 (2.3%)	9 (64.3%)	11 (42.3%)	456 (69.9%)	481 (52.9%)
Missing	1 (0.5%)	1 (7.1%)	0 (0%)	162 (24.8%)	164 (18.0%)
PPV	211/217 = 97.2%	4/14 = 28.6%	15/26 = 57.7%		
NPV		10/14 = 71.4%	11/26 = 42.3%	618/652 = 94.8%	

Percentages in parentheses are column-based and within each comparator category, IHC or ISH. For the purpose of this analysis, we did not define a corresponding group 4 borderline/equivocal group. *PPV* positive predictive value, *NPV* negative predictive value.

### ERBB2 SNP rs1058808

The single nucleotide polymorphism (SNP) rs1058808 was included in the assay as a genotypic marker and because this SNP has previously been suggested to be related to HER2 protein expression and risk for cancer^24^, as well as increased risk for trastuzumab-induced cardiotoxicity^25^. However, in our data rs1058808 genotype had no correlation to any standard clinicopathological variables (age, lymph nodes, tumor size, hormone receptors, or grade), clinical HER2 IHC score or HER2 status, nor to ERBB2 CN or ERBB2/CEP17 ratio. Of note, plotting heterozygous ERBB2 cases by genotype confirmed that all cases of ERBB2 amplification are monoallelic (either gains of the G or C allele, rarely both; Supplementary Fig. 2).

### ERBB2 CN survival analyses

**Fig. 4:**
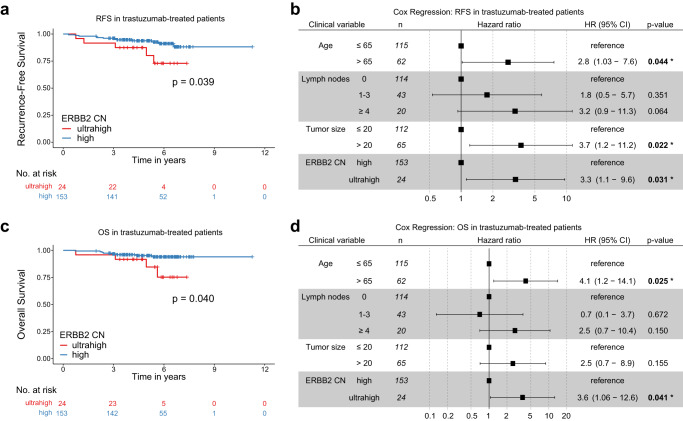
Kaplan–Meier survival analysis and forest plots of multivariable analyses of patients with HER2-amplified primary breast cancer treated with adjuvant trastuzumab (no neoadjuvant therapy), dichotomized by ddPCR into ERBB2-amplified or ultrahigh ERBB2-amplified (top 13.6% ERBB2 CN). **a**, **b** Ultrahigh ERBB2 CN predicts significantly worse recurrence-free survival (RFS) and is also an independent factor. **c**, **d** Ultrahigh ERBB2 amplification predicts significantly worse overall survival (OS) and is also an independent factor. The log-rank test was used for Kaplan–Meier survival analyses. Forest plots present the hazard ratio (square) and 95% confidence intervals (CI); *Y*-axis scale is log2 distance. Significant multivariable Cox regression *p*-values are indicated by asterisk. CN copy number.

We were next interested in investigating the relationship between ERBB2 CN and patient outcomes. We focused on ERBB2 CN as this variable best represents the total average “dose” of ERBB2 in a tumor and also because it showed slightly superior classification performance (Table 2). Within the 909 primary breast cancer cohort, 177 patients received adjuvant trastuzumab alone or in combination with chemotherapy and/or endocrine treatment. Using the ‘survminer’ R package, the 177 patients were dichotomized into two groups based on the identified ERBB2 CN cut-off value of 19.7, with cases above CN 19.7 termed biologically “ultrahigh” ERBB2 CN (24 cases, or 13.6%), and cases below this termed classic “high” ERBB2 CN (153 cases, or 86.4%). In this cohort with median 5.4 years follow-up time, Kaplan–Meier analysis revealed that patients with ultrahigh ERBB2 CN had significantly worse recurrence-free survival (RFS; *p* = 0.039, log-rank test) and overall survival (OS; *p* = 0.040, log-rank test) (Fig. 4a, c). Moreover, multivariable Cox regression analysis including all clinical variables significant in any univariable analysis demonstrated ERBB2 CN to be an independent biomarker for poor outcome in trastuzumab-treated primary breast cancer with a hazard ratio (HR) of 3.3 (95% CI 1.1–9.6; *p* = 0.031) for RFS and HR 3.6 (95% CI 1.1–12.6; *p* = 0.041) for OS (Fig. 4b, d). To note, all 24 ultrahigh ERBB2 CN status cases were clinically Ki67 high (*p* = 0.016, chi-square test), but there was no association to any other clinicopathological variables (age, lymph nodes, tumor size, hormone receptors, or grade).

**Fig. 5:**
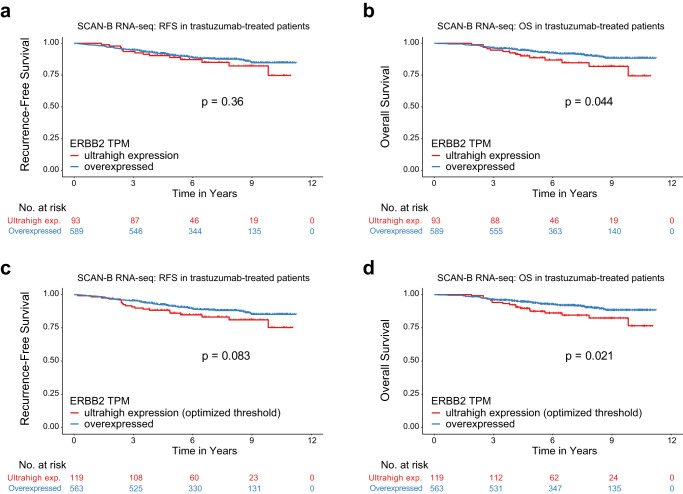
Kaplan-Meier survival analysis of the RNA-seq-based ultrahigh ERBB2 classification in an extended cohort of 682 consecutive patients with primary breast cancer, diagnosed between 2010 and 2018 and treated with adjuvant trastuzumab, selected from SCAN-B. To mirror the ddPCR proportion, the ultrahigh ERBB2 mRNA classification was based on the top 13.6% cases with highest overexpression of ERBB2 transcripts per million mapped reads (TPM) as measured by RNA-seq and related to (**a**) recurrence-free survival (RFS) and (**b**) overall survival (OS). Since ERBB2 mRNA is a surrogate to ERBB2 CN, a secondary analysis was performed to obtain the optimal threshold for ERBB2 mRNA, which increased the ultrahigh group to 17.4%, and obtained greater separation of the (**c**) RFS and (**d**) OS curves. Kaplan–Meier *p*-values were calculated using the log-rank test.

To expand upon and further validate this result, we sought a larger cohort of patient material treated with trastuzumab. It was infeasible to obtain DNA material from a large series of such breast cancers, therefore we turned to the RNA-seq data from within the SCAN-B study (ClinicalTrials.gov NCT02306096). We retrieved 682 consecutive HER2-positive primary breast cancer patients from SCAN-B diagnosed between 2010 and 2019 and who had received adjuvant trastuzumab and had available RNA-seq data. The median follow-up time for this cohort was 6.8 years. To mirror the ultrahigh ERBB2 CN group, we selected an identical proportion of 13.6% of cases with the highest ERBB2 mRNA expression levels. Similar to our observations in the ddPCR cohort, we found in the RNA-seq cohort that patients with ultrahigh ERBB2 expression had a significantly worse OS (*p* = 0.044, log-rank test) but not RFS (Fig. 5a, b). Since ERBB2 mRNA expression is only a surrogate for DNA CN, we investigated whether an mRNA-specific threshold may be more relevant for defining an ultrahigh group by RNA-seq. This optimization analysis resulted in an enlargement of the ultrahigh group to 17.5%, and now even more in line with our results from ddPCR, showed that cases with ultrahigh ERBB2 mRNA trended towards inferior RFS (*p* = 0.083, log-rank test) and had a significantly worse OS (*p* = 0.021, log-rank test) (Fig. 5c, d). For this optimized classification, multivariable analysis showed that ≥ 4 positive lymph nodes was significant for RFS (*p* = 0.001, multivariable Cox regression) and that both ≥ 4 positive lymph nodes and ERBB2 ultrahigh were significant factors for OS (multivariable Cox regression *p* = 0.001 and *p* = 0.039, respectively).

## Discussion

Reliable testing of HER2 status is critically consequential for guiding treatment decisions for HER2 targeted therapy and optimizing clinical outcome in BC patients. The current guidelines recommend testing all invasive breast cancers for HER2, typically with IHC in combination with FISH when necessary, especially in IHC 2+ cases. However, there are known limitations for both methods. In this study, we developed a multiplex ddPCR assay utilizing one probe for the ERBB2 gene locus, one centromeric control probe (CEP17), and a copy number stable control region located near cytoband 2p13.1 (CNS-2p13.1) to quantify ERBB2 amplification. With this assay, both average ERBB2 signals/genome and ERBB2/CEP17 ratios can be calculated in an analogous way as dual probe ISH. In both training and validation groups, our results demonstrated high concordance of HER2 status determined by ddPCR with clinical HER2 IHC and ISH status, and we found that the copy number stable control region performed slightly better than CEP17 to define HER2 status. Interestingly, we identified an ERBB2 ultrahigh amplification group that had worse survival on adjuvant trastuzumab than patients with moderate ERBB2 amplification. Although not evaluated in this study, our ddPCR assay design with very short amplicons (≤59 bp) should be highly amenable to analysis of ERBB2 CN in other sample types including those where the DNA may be highly fragmented such as formalin-fixed paraffin embedded tissues (FFPE; <600 bp) as well as circulating tumor DNA (ctDNA; <200 bp) liquid biopsy samples.

Although ddPCR has been used previously to measure HER2 copy number^14,26^, all studies used either CEP17 or a non-chromosome 17 reference locus and not both^12,14–20,26^. However, using either reference alone may misclassify HER2 status for cases with co-amplification of CEP17 or monosomy 17. For example, one study reported 83.3% (5/6) co-amplified and 21.1% (4/19) monosomy 17 cases by ISH were clinically HER2 positive^13^. This issue is not negligible, as CEP17 copy numbers >3 was found in 6.7% of cases in our study and has been reported in up to 8% of cases by FISH in breast cancer^27,28^. It was previously believed to be polysomy of chromosome 17, but is now believed as gain or amplification of the centromeric region. Thus using CEP17 alone might not be an optimal ddPCR reference probe for HER2 diagnostics. In this study, 57.7% (15/26) co-amplified cases were HER2 positive, which means that 15/264 HER2 positive cases could be misclassified as HER2 negative when CEP17 is used as the only reference. Similarly, 28.6% (4/14) monosomy 17 cases were HER2 positive. Thus, 4/264 HER2 positive cases could be misclassified as HER2 negative when only using the other reference. The proportions of HER2 positive cases in co-amplification and monosomy 17 groups appeared to be reasonable and could explain why CNS-2p13.1 is superior to CEP17 as reference.

We and others have shown that HER2 status determined by ddPCR has high accuracy. Our in-house reagent and consumables cost per ERBB2 CN ddPCR reaction is estimated to be less than 10 EUR. Moreover, the labor for ddPCR setup and data analysis are highly automatable with minimal hands-on time or subjective steps. These all are factors which compare favorably to IHC and ISH. However, even though ddPCR has advantages of being more rapid, less labor-intensive, less expensive, and more reproducible, ddPCR is not ready to replace IHC or ISH. In this study, the PPV for group 1 classic HER2 amplification was 97.2%. There were 6 cases with ERBB2 CN ≥ 3.05 and ERBB2/CEP17 ≥ 1.55, but HER2 negative based on clinical IHC/ISH. We hypothesize that these discrepancies may be due to tumor heterogeneity, laboratory error, or false-negative low protein with no ISH performed. Past studies have reported between 1.5–5% of IHC 0/1+ can be ISH-amplified^29–32^.

The association of ERBB2 amplification level and outcome after trastuzumab-based treatment has been debated in the literature, with various testing methods, sample types, follow-up time, and classification thresholds (see e.g. review and meta-analysis by Xu et al. ^33^). For example, in one of the largest prior studies, 1703 HER2-positive cases were divided based on HER2 FISH ratio levels or HER2 FISH CN levels and found no significant influence of the degree of HER2 amplification on disease-free survival after 1-year of adjuvant trastuzumab after a median 2-years of follow-up in early stage BC patients treated with prior adjuvant chemotherapy^34^. This being said, they did note a trend for decreased benefit with increasing HER2 FISH ratio which was less evident when HER2 was quantified by HER2 FISH CN only. In this previous study, one difference is that they divided all patients into quartiles, thus approximately 25% of patients had HER2 FISH scored in the upper most category, CN > 18, which is a larger ultrahigh group than in our analyses. Another difference is they only looked at disease-free survival, and had a relatively short follow-up time. In our study, the separation of Kaplan-Meier survival curves emerges after about 2–3 years post-surgery; therefore, the longer-term survival difference may have been missed in this earlier study with median of 2 years follow-up. This delayed survival separation is intriguing and may suggest that ongoing anti-HER2 treatment can stabilize disease within the ERBB2 ultrahigh group, but after cessation of trastuzumab, formerly controlled clones can proliferate again. Furthermore, we see a stronger survival difference for OS than for RFS. Of note, as ASCO-CAP guidelines recommend to count a minimum of 20 non-overlapping cells per slide for FISH, ddPCR scoring is much less subjective and labor intensive.

On the other hand, another study found that HER2-positive BC patients with very high HER2 protein content may benefit less from adjuvant trastuzumab compared with those whose cancer has more moderate HER2 content^35^. In that study of 196 patients, 13% of cases belonged to the ultrahigh HER2 protein group, a proportion of patients similar to our result with 13.6% with ultrahigh ERBB2 CN in the present study. The fact that our survival association was corroborated even using RNA-seq gene expression analysis as a surrogate for ultrahigh ERBB2 strongly suggests that increased ERBB2 CN may be associated to decreased benefit to adjuvant trastuzumab in primary BC.

Our study has a number of limitations. Intratumoral heterogeneity and tumor cell content pose challenges for ddPCR when tissues are processed for DNA: for example when tumor cell content is low in a sample, the DNA CN may be artificially compressed towards normal CN due to an abundance of non-tumor cells with normal CN. Despite this potential confounder, tumor cell content was previously evaluated and available for 579 cases within the 909 BC cohort^36^ and was not significantly associated to the rates of HER2 status false-negatives, false-positives, true-negatives, or true-positives in our material (Supplementary Fig. 3). In addition to issues of cellularity and heterogeneity, discordances to clinical HER2 may be due to ddPCR laboratory procedures and/or clinical laboratory performance. The associations to outcome are highly interesting but should be replicated. To note, the available SCAN-B clinical follow-up information, in particular for RFS, is somewhat sparse at present due to a number of factors including those related to the COVID-19 pandemic. The trend, but not significant association, of ultrahigh ERBB2 mRNA to poorer RFS that we report herein may be due to partially inadequate clinical follow-up information available today. Subsequent re-analysis is warranted when even longer follow-up information has accrued and been collected. Furthermore, our study does not address “HER2-low” breast cancer, a potentially large subgroup of BC that is poorly defined^37^ as having low to moderate levels of HER2 expression (always ISH-negative but either IHC 1+ or IHC 1+ / 2+) and that has been shown to generally respond to antibody-drug conjugates such as trastuzumab-deruxtecan (T-DXd) in the metastatic BC setting^38^. As expected, a ddPCR assay for DNA CN is poorly informative for delineating a HER2-low group (data not shown), whereas a ddPCR assay for quantification of ERBB2 expression levels using RNA as input may be much more relevant.

In conclusion, our results show the high promise for ddPCR to aid in clinical diagnostics for HER2 CN evaluation and the benefits of utilizing more than one reference locus. The high PPV for classic group 1 HER2 amplification may suggest that an upfront ddPCR test may be a rapid and cost-effective screen prior to or in parallel with IHC, and before confirmation by ISH only for challenging cases. The biological ultrahigh ERBB2 group is interesting, and if corroborated in additional future studies, clinical trials may be warranted to evaluate whether additional or alternative systemic treatment can achieve parity in longer-term outcomes for ultrahigh ERBB2 cases as seen for patients with moderate amplification.

## Methods

### Ethics and clinical cases

The study was approved by the Regional Ethical Review Board of Lund at Lund University (approval numbers 2009/658, 2010/383, 2012/58, 2013/459, and 2015/277) and performed in accordance with the Declaration of Helsinki. For all cohorts, health professionals provided patient information and patients gave written informed consent. The ddPCR patient cohort contained a total of 909 primary invasive breast tumors selected as follows: 510 cases consisting of three random selections of 170 BCs each from within the clinical HER2 IHC 0-1+, 2+, and 3+ groups^36^, plus 399 of 405 cases previously described^39^. The patients were diagnosed between 2006 to 2019 and were treated at the Skåne University Hospital in Malmö and Lund and tumor tissue from primary surgery were flash frozen. Clinicopathological data were retrieved from the Swedish National Breast Cancer Registry (NKBC) and published results^39^. HER2 IHC and ISH status were evaluated as described^39^. Of the 909 patients, 177 patients were clinically HER2 positive, received no neoadjuvant treatment, and received adjuvant trastuzumab in combination with chemotherapy and/or endocrine therapy according to national treatment guidelines. For validation using RNA-seq data, for 682 consecutive patients from the SCAN-B cohort diagnosed between 2010 and 2018 with HER2 positive primary breast cancer, receiving no neoadjuvant therapy and treated with adjuvant trastuzumab in combination with chemotherapy and/or endocrine therapy, the corresponding gene expression data and survival information were retrieved for the tumor specimen obtained at surgery^40^.

### DNA preparation

DNA was isolated from fresh tumor samples obtained at primary surgery as previously described^36,41^. The DNA concentration was determined by a NanoDrop 2000 spectrophotometer (Thermo Scientific, Waltham, Massachusetts, USA). The positive control ERBB2-amplified SK-BR-3 breast cancer cell line was obtained from ATCC/LGC, and DNA was isolated from a low passage culture using the DNA Blood Mini Kit (Qiagen, Hilden, Germany). NS12911, the human reference genetic material repository DNA sample, was purchased from Coriell Institute for Medical Research (Camden, New Jersey, USA).

### Droplet digital PCR

A single-reaction multiplex dPCR assay was developed that enables simultaneous quantification of two common alleles of *ERBB2*, a control region CEP17 (a locus within the chromosome 17 centromere) and a CN stable control region^42^ located near cytoband 2p13.1 (CNS-2p13.1) (Fig. 1a). The PCR products have the sequences CCT GCT GGT GCC ACT CTG GAA AGG CCC AAG ACT CTC TCC CCA GGG AAG AAT GGG GTC GT for ERBB2 (59 bp), TTC CTG CAG CCC TTG ACT GGG CTG GAC CTG CTG CCC CAG GTA CGT GTT ACA GTG CAG GA for CEP17 (59 bp), and CGG GTC TTC ATG CCG AAG TAG ACG TGA GGG CGG TAG GTT CCC CAG AAG AGG TCC GGG G for CNS-2p13.1 (58 bp), respectively. Digital PCR was performed with positive control, negative control and no template control within each run. Briefly, a total 20 µl PCR reaction was prepared with 10 ng DNA and 4X ddPCR Supermix (Bio-Rad, Hercules, California, USA) following manufacturer instructions. Droplets were generated using a Bio-Rad Automated Droplet Generator, and the emulsified droplet reactions were transferred to a 96-well plate and heat sealed before thermocycling using a Bio-Rad T100 Thermal Cycler with the following protocol: 95 °C for 10 min, 40 cycles of 94 °C for 30 s and 60 °C for 60 s, 98 °C for 10 min, and hold at 4 °C. The temperature ramp rate was 2 °C/s for all steps. After PCR, the plates were read using a Bio-Rad QX200 Droplet Reader. Analysis of ddPCR data was performed using QuantaSoft v1.3.2.0 software from Bio-Rad. Nine runs for each positive control SK-BR-3 and negative control human normal genome NS12911 were performed, each on a different date and/or operator. Across all experiments in this study, a mean of 19738 droplets were analyzed per reaction (SD 3491).

### RNA-sequencing

The Sweden Cancerome Analysis Network Breast Initiative (SCAN-B; ClinicalTrials.gov identifier NCT02306096) is an ongoing population-based multicenter study covering a wide geography of Sweden that has, to date, prospectively enrolled more than 20,000 patients with BC and performed RNA-sequencing on more than 15,000 breast tumors. Details of the study and sample preparation and sequencing protocols have been described previously^36,43–45^, the and the ERBB2 gene expression measurements were normalized between library preparation protocols as previously described^40^. In brief, gene expression TPM transcripts per million (TPM) measurements were generated using StringTie v1.3.3b as described previously^45^. An offset of 0.1 was added to all expression measurements (to avoid logarithm of zero) followed by log2 transformation, then batch correction normalization per gene (ERBB2) between library preparation protocols (dUTP, TruSeq, and NeoPrep) was performed by calculating a protocol-wise conversion factor using the mean expression within each protocol, and centering using the mean from dUTP as the reference baseline^40^.

### Data and statistical analyses

Prior to data analysis, the 909 patient cohort was randomly divided into 2 groups: training group (70%; 636 cases) and validation group (30%; 273 cases) (Fig. 1c). ERBB2 CN was calculated as 2 * ERBB2 copies / CNS-2p13.1 copies tested by ddPCR per reaction, with the assumption that CNS-2p13.1 CN is 2, and the ERBB2/CEP17 ratio was calculated as ERBB2 copies / CEP17 copies. Thresholds to determine HER2 status by ddPCR were optimized using receiver operating characteristic curve analysis which was performed using the ‘pROC’ package in R. Optimal sensitivity and specificity, or maximum performance, was defined as the maximum Youden’s index (sensitivity + specificity − 1). For statistical correlations, continuous variables were compared using Student’s t-test and categorical variables were compared using Chi-square test or Fisher’s exact test. Optimal dichotomization of ERBB2 CN or ERBB2 TPM was determined using the ‘surv_cutpoint’ function of the ‘survminer’ R package. Using the package ‘survival’, survival curves were estimated using the Kaplan-Meier method and compared using the log-rank test, and hazard ratios calculated by univariable and multivariable Cox regression analyses. The following standard variables were considered in univariable analysis: age, lymph nodes, tumor size, estrogen receptor status, progesterone receptor status, Ki67 status, and grade. For multivariable Cox regression, all variables with *p* < 0.1 in any univariable analysis for either RFS or OS were included as co-variates in multivariable analysis for both RFS and OS, and the Schoenfeld residual test was performed to verify the assumption of proportional hazards. All statistical tests were two-tailed and *p*-values < 0.05 were considered significant.

### Reporting summary

Further information on research design is available in the Nature Research Reporting Summary linked to this article.

### Supplementary information

Supplementary Information

Reporting Summary

## Data Availability

ERBB2 gene expression data and associated clinicopathological information utilized in this study are available from Dryad Digital Repository (10.5061/dryad.rv15dv4dm). Raw sequencing data is regarded as personal information by Swedish law and cannot be made publicly accessible.

## Code availability

All analyses were performed in R version 4.2. The R packages ‘ggplot2’, ‘pROC’, ‘surv_cutpoint’, ‘survminer’, and ‘survival’ were used as described above. The custom code used is available at https://github.com/translational-oncogenomics/HER2_CNA_ddPCR.
