# Supplementary material for: Digital PCR quantification of ultrahigh ERBB2 copy number identifies poor breast cancer survival after trastuzumab
Source: NPJ Breast Cancer. 2024 Feb 19;10:14. doi: 10.1038/s41523-024-00621-x (PMC10876644; doi:10.1038/s41523-024-00621-x)
Supplement: Supplementary file 1 — Supplementary Information [file 41523_2024_621_MOESM1_ESM.pdf]

SUPPLEMENTARY INFORMATION

Digital PCR quantification of ultrahigh ERBB2 copy number identifies poor breast cancer survival after trastuzumab

Pei Meng[1#], Hina Dalal[1#], Yilun Chen[2], Christian Brueffer[1,2], Sergii Gladchuk[1,2], Miguel Alcaide[2], Anna Ehinger[1,3], and Lao H. Saal[1*]

[1] Division of Oncology, Department of Clinical Sciences Lund, Lund University, Medicon Village, SE-22381, Lund, Sweden.

[2] SAGA Diagnostics AB, Scheelevägen 2, MV406, SE-22381 Lund, Sweden.

[3] Department of Genetics and Pathology, Laboratory Medicine, Region Skåne, Lund, Sweden.

[#] These authors contributed equally: Pei Meng, Hina Dalal

[*] Corresponding author: Lao.Saal@med.lu.se

Supplementary Information contains Supplementary Figures 1-3.

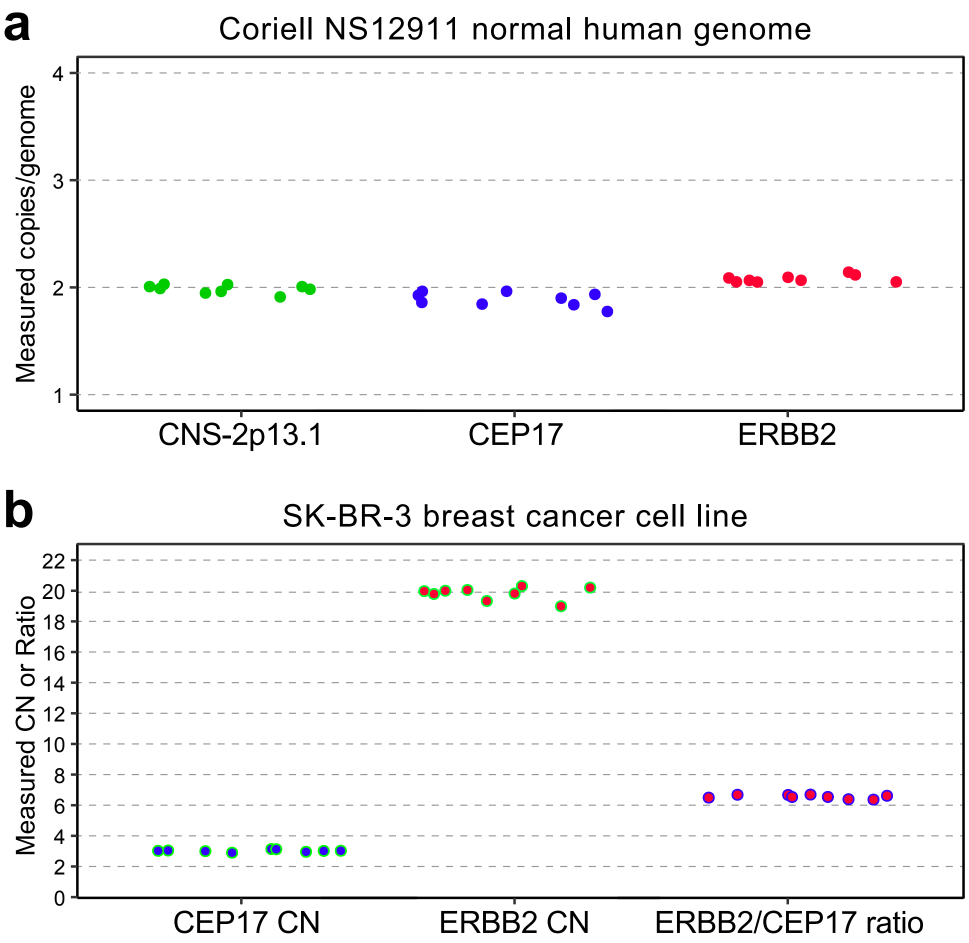

Supplementary Figure 1. ERBB2 ddPCR multiplex assay results for replicate analyses of **a** Coriell NS12911 normal human genomic DNA (n=9) and **b** ERBB2-amplified SK-BR-3 positive control breast cancer cell line (n=9) demonstrate high reproducibility for quantification of CNS-2p13.1, CEP17, ERBB2, CEP17 CN, ERBB2 CN, and ERBB2/CEP17 ratio. Each replicate analysis was performed on a different date and/or operator. CN = copy number.

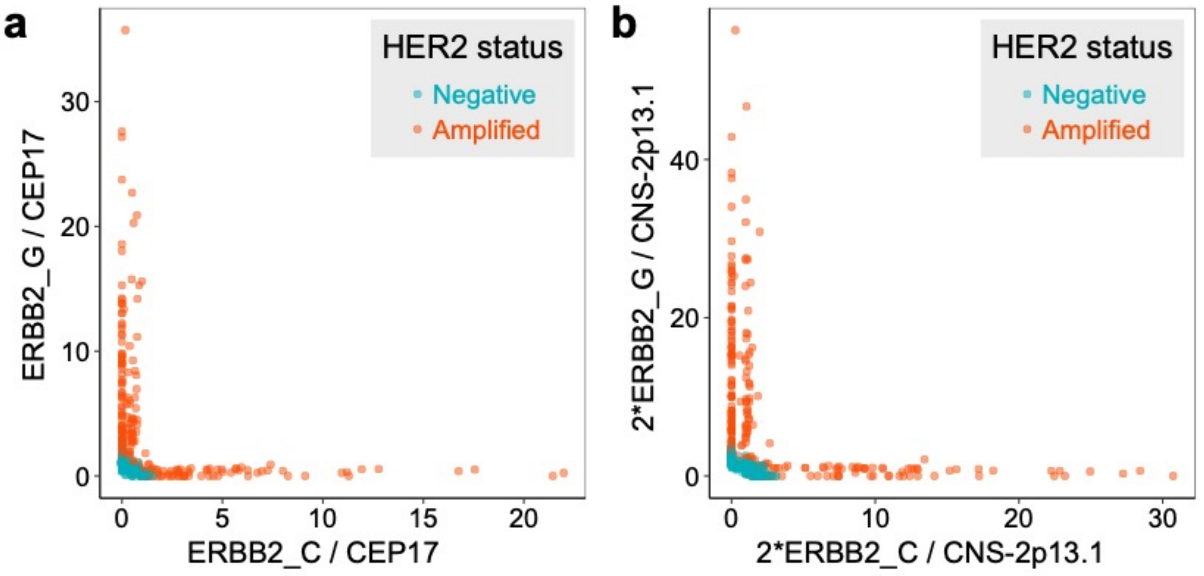

Supplementary Figure 2. ERBB2 rs1058808 SNP genotype (G or C allele) confirms monoallelic amplification in all informative (heterozygous) cases. **a** Allele plot by ERBB2_allele/CEP17 ratio, and **b** plot by ERBB2_allele/CNS-2p13.1 ratio. Clinical HER2 status is indicated by the marker color.

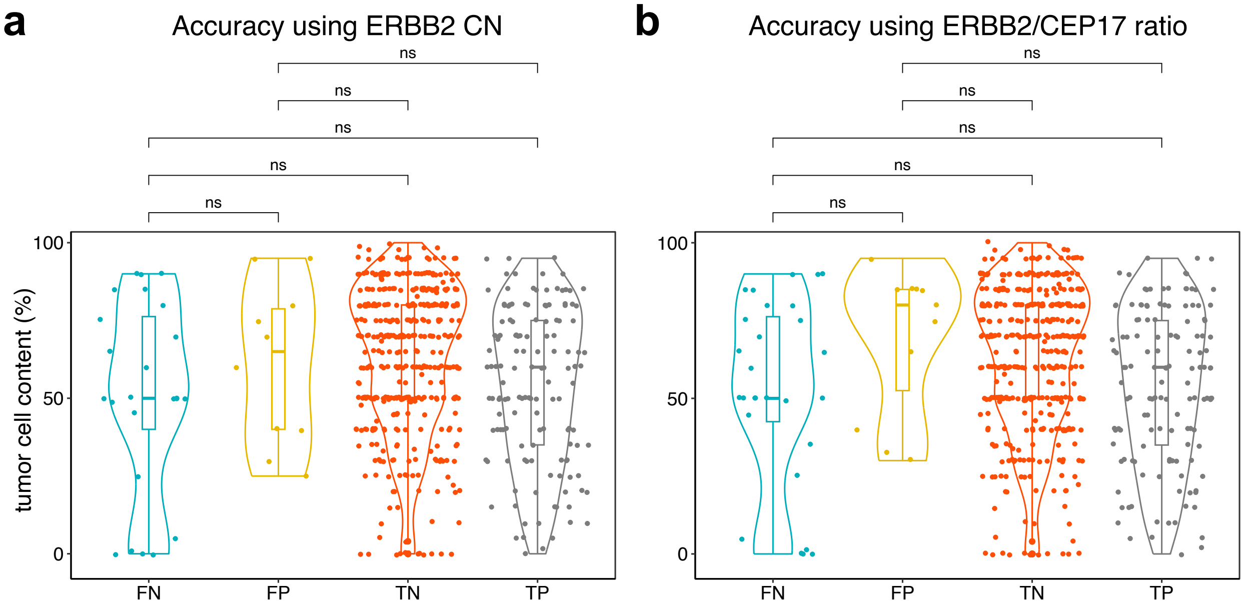

Supplementary Figure 3. For 579 cases with available tumor cell content information (obtained from a tissue sample cut immediately adjacent from the sample processed and analyzed by ddPCR), the percentage of tumor cells in the sample has no significant association to the rates of false-negatives (FN), false-positives (FP), true-negatives (TN), or true-positives (TP) using either the **a** ERBB2 CN or **b** ERBB2/CEP17 ratio. ns = not significant.
