# Supplementary material for: Digital PCR quantification of ultrahigh ERBB2 copy number identifies poor breast cancer survival after trastuzumab
Source: NPJ Breast Cancer. 2024 Feb 19;10:14. doi: 10.1038/s41523-024-00621-x (PMC10876644; doi:10.1038/s41523-024-00621-x)
Supplement: Supplementary file 2 — Reporting Summary [file 41523_2024_621_MOESM2_ESM.pdf]

nature portfolio

Corresponding author(s): Saal, LH

Last updated by author(s): Jan 30, 2024

Reporting Summary

Nature Portfolio wishes to improve the reproducibility of the work that we publish. This form provides structure for consistency and transparency in reporting. For further information on Nature Portfolio policies, see our Editorial Policies and the Editorial Policy Checklist.

Statistics

For all statistical analyses, confirm that the following items are present in the figure legend, table legend, main text, or Methods section.

n/a	Confirmed	
☐	☒	The exact sample size (*n*) for each experimental group/condition, given as a discrete number and unit of measurement
☐	☒	A statement on whether measurements were taken from distinct samples or whether the same sample was measured repeatedly
☐	☒	The statistical test(s) used AND whether they are one- or two-sided<br>*Only common tests should be described solely by name; describe more complex techniques in the Methods section.*
☐	☒	A description of all covariates tested
☐	☒	A description of any assumptions or corrections, such as tests of normality and adjustment for multiple comparisons
☐	☒	A full description of the statistical parameters including central tendency (e.g. means) or other basic estimates (e.g. regression coefficient) AND variation (e.g. standard deviation) or associated estimates of uncertainty (e.g. confidence intervals)
☐	☒	For null hypothesis testing, the test statistic (e.g. *F*, *t*, *r*) with confidence intervals, effect sizes, degrees of freedom and *P* value noted<br>*Give P values as exact values whenever suitable.*
☒	☐	For Bayesian analysis, information on the choice of priors and Markov chain Monte Carlo settings
☒	☐	For hierarchical and complex designs, identification of the appropriate level for tests and full reporting of outcomes
☒	☐	Estimates of effect sizes (e.g. Cohen's *d*, Pearson's *r*), indicating how they were calculated

Our web collection on statistics for biologists contains articles on many of the points above.

Software and code

Policy information about availability of computer code

Data collection	QuantaSoft v1.3.2.0 software from Bio-Rad.
Data analysis	All analyses were performed in R version 4.2, and the following packages were utilized: ggplot2, pROC, surv_cutpoint, survminer, and survival. Custom code is provided at GitHub (https://github.com/translational-oncogenomics/HER2_CNA_ddPCR).

For manuscripts utilizing custom algorithms or software that are central to the research but not yet described in published literature, software must be made available to editors and reviewers. We strongly encourage code deposition in a community repository (e.g. GitHub). See the Nature Portfolio guidelines for submitting code & software for further information.

Data

Policy information about availability of data

All manuscripts must include a data availability statement. This statement should provide the following information, where applicable:

- Accession codes, unique identifiers, or web links for publicly available datasets
- A description of any restrictions on data availability
- For clinical datasets or third party data, please ensure that the statement adheres to our policy

ERBB2 gene expression data and associated clinicopathological information utilized in this study are available from Dryad Digital Repository (https://doi.org/10.5061/dryad.rv15dv4dm). Raw sequencing data is regarded as personal information by Swedish law and cannot be made publicly accessible.

Research involving human participants, their data, or biological material

Policy information about studies with human participants or human data. See also policy information about sex, gender (identity/presentation), and sexual orientation and race, ethnicity and racism.

Reporting on sex and gender	The study is of human breast cancer which is almost entirely a disease of women with very few diagnoses within men. Neither sex, gender, or sexual orientation were utilized in our study.
Reporting on race, ethnicity, or other socially relevant groupings	Race, ethnicity, or other socially relevant groups were not utilized in our study.
Population characteristics	The population characteristics are described in Table 1. The ddPCR patient cohort contained a total of 909 primary invasive breast tumors selected as follows: 510 cases consisting of three random selections of 170 BCs each from within the clinical HER2 IHC 0-1+, 2+, and 3+ groups, plus 399 of 405 cases previously described. The patients were diagnosed between 2006 to 2019 and were treated at the Skåne University Hospital in Malmö and Lund and tumor tissue from primary surgery were flash frozen. Of the 909 patients, 177 patients were clinically HER2 positive, received no neoadjuvant treatment, and received adjuvant trastuzumab in combination with chemotherapy and/or endocrine therapy according to national treatment guidelines. For validation using RNA-seq data, 682 consecutive patients from the SCAN-B cohort diagnosed between 2010 and 2018 with HER2 positive primary breast cancer, receiving no neoadjuvant therapy and treated with adjuvant trastuzumab in combination with chemotherapy and/or endocrine therapy, were selected.
Recruitment	Patients were recruited to the study as a subselection from within the population-based study SCAN-B. The Sweden Cancerome Analysis Network Breast Initiative (SCAN-B; ClinicalTrials.gov identifier NCT02306096) is an ongoing population-based multicenter study covering a wide geography of Sweden that has, to date, prospectively enrolled more than 20,000 patients with BC and performed RNA-sequencing on more than 15,000 breast tumors.
Ethics oversight	The study was approved by the Regional Ethical Review Board of Lund at Lund University (approval numbers 2009/658, 2010/383, 2012/58, 2013/459, and 2015/277) and performed in accordance with the Declaration of Helsinki.

Note that full information on the approval of the study protocol must also be provided in the manuscript.

Field-specific reporting

Please select the one below that is the best fit for your research. If you are not sure, read the appropriate sections before making your selection.

☒ Life sciences ☐ Behavioural & social sciences ☐ Ecological, evolutionary & environmental sciences

For a reference copy of the document with all sections, see nature.com/documents/nr-reporting-summary-flat.pdf

Life sciences study design

All studies must disclose on these points even when the disclosure is negative.

Sample size	Large cohorts meeting specific criteria were selected in order to strengthen the generalizability of the results. The ddPCR patient cohort contained a total of 909 primary invasive breast tumors selected as follows: 510 cases consisting of three random selections of 170 BCs each from within the clinical HER2 IHC 0-1+, 2+, and 3+ groups, plus 399 of 405 cases previously described. The patients were diagnosed between 2006 to 2019 and were treated at the Skåne University Hospital in Malmö and Lund and tumor tissue from primary surgery were flash frozen. Of the 909 patients, 177 patients were clinically HER2 positive, received no neoadjuvant treatment, and received adjuvant trastuzumab in combination with chemotherapy and/or endocrine therapy according to national treatment guidelines. For validation using RNA-seq data, 682 consecutive patients from the SCAN-B cohort diagnosed between 2010 and 2018 with HER2 positive primary breast cancer, receiving no neoadjuvant therapy and treated with adjuvant trastuzumab in combination with chemotherapy and/or endocrine therapy, were selected. Although no power calculations were performed, these large sample sizes are thought to be sufficient given how they were selected.
Data exclusions	No data were excluded from analyses.
Replication	The primary ddPCR results were validated using RNA-seq data and similar findings are reported.
Randomization	Prior to data analysis, the 909 patient cohort was randomly divided into 2 groups only once: training group (70%; 636 cases) and validation group (30%; 273 cases). Tumor samples were categorized by clinicopathological information. The following standard variables were considered in univariable analysis: age, lymph nodes, tumor size, estrogen receptor status, progesterone receptor status, Ki67 status, and grade. For multivariable Cox regression, all variables with $p<0.1$ in any univariable analysis for either RFS or OS were included as co-variates in multivariable analysis for both RFS and OS, and the Schoenfeld residual test was performed to verify the assumption of proportional hazards.
Blinding	It was not possible to blind the investigators to the clinicopathological information. All raw data was generated without any consideration of any clinicopathological information. Random division into the training group and validation group was performed only once. The validation group was not utilized until all training group analyses were completed. The validation group was evaluated only once. For validation using RNA-seq data, a consecutive series of all patients, from within a population-based cohort SCAN-B, that met specific criteria within a diagnosis time period were selected. Thus, potential sources of bias were adequately controlled.

Reporting for specific materials, systems and methods

We require information from authors about some types of materials, experimental systems and methods used in many studies. Here, indicate whether each material, system or method listed is relevant to your study. If you are not sure if a list item applies to your research, read the appropriate section before selecting a response.

Materials & experimental systems

n/a	Involved in the study
☒	☐ Antibodies
☐	☒ Eukaryotic cell lines
☒	☐ Palaeontology and archaeology
☒	☐ Animals and other organisms
☐	☒ Clinical data
☒	☐ Dual use research of concern
☒	☐ Plants

Methods

n/a	Involved in the study
☒	☐ ChIP-seq
☒	☐ Flow cytometry
☒	☐ MRI-based neuroimaging

Eukaryotic cell lines

Policy information about cell lines and Sex and Gender in Research

Cell line source(s)	The positive control ERBB2-amplified SK-BR-3 breast cancer cell line was obtained from ATCC/LGC (Teddington, UK). NS12911, the human reference genetic material repository DNA sample, was purchased from Coriell Institute for Medical Research (Camden, New Jersey, USA).
Authentication	Cells were grown only to low passage (<10) before isolation of DNA; therefore, further authentication was not performed. The ERBB2 copy number was measured and reflected expectations based on published third-party results.
Mycoplasma contamination	All cells tested negative for mycoplasma contamination.
Commonly misidentified lines (See ICLAC register)	None.

Clinical data

Policy information about clinical studies

All manuscripts should comply with the ICMJE guidelines for publication of clinical research and a completed CONSORT checklist must be included with all submissions.

Clinical trial registration	*Provide the trial registration number from ClinicalTrials.gov or an equivalent agency.*
Study protocol	*Note where the full trial protocol can be accessed OR if not available, explain why.*
Data collection	*Describe the settings and locales of data collection, noting the time periods of recruitment and data collection.*
Outcomes	*Describe how you pre-defined primary and secondary outcome measures and how you assessed these measures.*

Plants

Seed stocks	*Report on the source of all seed stocks or other plant material used. If applicable, state the seed stock centre and catalogue number. If plant specimens were collected from the field, describe the collection location, date and sampling procedures.*
Novel plant genotypes	*Describe the methods by which all novel plant genotypes were produced. This includes those generated by transgenic approaches, gene editing, chemical/radiation-based mutagenesis and hybridization. For transgenic lines, describe the transformation method, the number of independent lines analyzed and the generation upon which experiments were performed. For gene-edited lines, describe the editor used, the endogenous sequence targeted for editing, the targeting guide RNA sequence (if applicable) and how the editor was applied.*
Authentication	*Describe any authentication procedures for each seed stock used or novel genotype generated. Describe any experiments used to assess the effect of a mutation and, where applicable, how potential secondary effects (e.g. second site T-DNA insertions, mosiacism, off-target gene editing) were examined.*
